# Fabrication of Morphology-Tailored ZIF-67/Polyether‑*b*‑Amide Mixed Matrix Membranes via CTAB-Assisted Hydrothermal Synthesis for CO_2_ and CO_2_/N_2_ Separation

**DOI:** 10.1021/acsomega.5c06995

**Published:** 2025-11-19

**Authors:** Paula S. Pacheco, Sônia F. Zawadzki, Daniel Eiras

**Affiliations:** a Graduate Program in Materials Engineering and Science (PIPE/UFPR), 28122 Federal University of Paraná, Jardim das Américas, Curitiba, Paraná 81530-000, Brazil; b Graduate Program in Chemistry (PPGQ/UFPR), 28122 Federal University of Paraná, Jardim das Américas, Curitiba, Paraná 81530-000, Brazil

## Abstract

The aim of this study was to evaluate the influence of particle morphology on the gas permeability and selectivity of poly­(ether-*block*-amide) (PEBAX MH-1657)/zeolitic imidazolate framework-67 (ZIF-67) mixed-matrix membranes. ZIF-67 particles were synthesized using cetyltrimethylammonium bromide (CTAB) as a morphology modulator, and membranes were fabricated via solution casting followed by solvent evaporation. Single-gas permeability and ideal selectivity for carbon dioxide/nitrogen and carbon dioxide/methane were measured using a variable volume/constant pressure setup with a capillary flowmeter, at 10 and 15 bar and 35 °C. The mixed-matrix membranes exhibited enhanced permeability and selectivity; at 10 bar, they surpassed the Robeson upper bound for CO_2_/N_2_ while approaching the bound for CO_2_/CH_4_. Maximum values obtained in this study were a CO_2_ permeability of 236 Barrer (NC 1%, 15 bar), a CO_2_/N_2_ selectivity of 110 (PL 5%, 10 bar), and a CO_2_/CH_4_ selectivity of 27 (PL 1%, 15 bar). Gas permeation results indicated that both pressure and ZIF-67 loading strongly influenced performance, with optimal behavior at 10 bar. Differential scanning calorimetry revealed morphology-dependent modifications of PEBAX crystallinity and glass transition temperature, which contributed to the observed permeability–selectivity trade-offs under different pressures.

## Graphical abstract

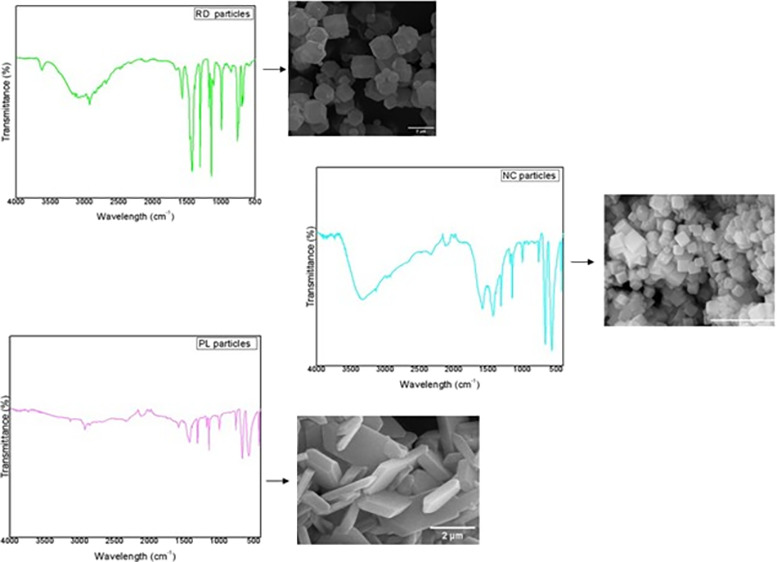

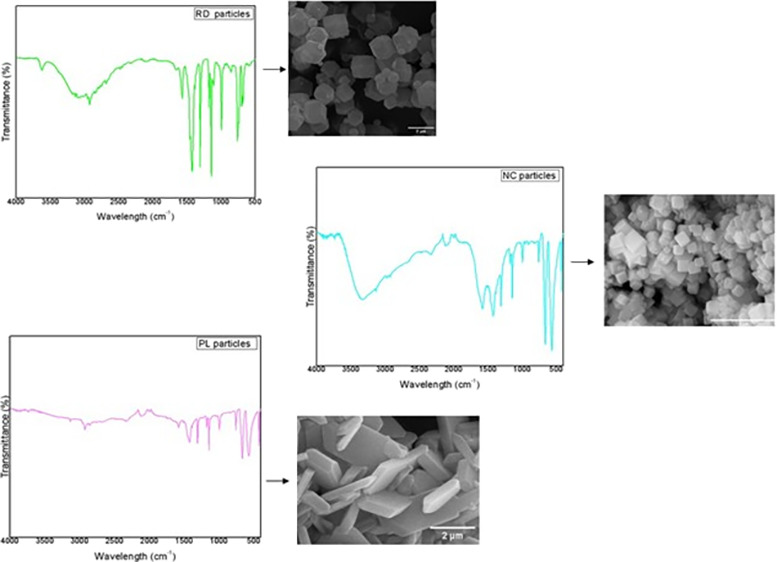

## Highlights

- ZIF-67 morphology and loading modulate MH-1657 thermal behavior, with *T*_g_ decreasing (down to −65.4 °C) and selective changes in PEO/PA crystallinity.
- These thermal modifications directly correlate with enhanced CO_2_ permeability (+26%) and selectivity (+39%) compared to neat Pebax.
- Hydrothermal synthesis improved particle dispersion and initial performance, while solvothermal synthesis ensured greater stability under high pressure (15 bar).

## 1 Introduction

Membrane technology has emerged as a key enabler in gas separation and carbon capture processes, offering effective solutions to global challenges such as climate change and the growing demand for cleaner energy sources. Membranes provide a sustainable, energy-efficient, and scalable alternative for separating gases such as CO_2_, N_2_, and CH_4_, and are widely implemented in sectors including natural gas purification and postcombustion CO_2_ capture.^1–3^ Among the various types of membranes, polymer-based membranes have attracted considerable attention due to their ease of processing, operational flexibility, and compatibility with existing industrial infrastructure. However, these membranes are often constrained by the well-known trade-off between permeability and selectivity, which limits their applicability in high-performance separation processes.^4,5^

To overcome this intrinsic limitation, mixed matrix membranes (MMMs) have emerged as a promising strategy. MMMs combine the favorable mechanical and processable properties of polymers with the high selectivity and permeability of inorganic fillers. The incorporation of materials such as zeolites, carbon nanotubes, graphene oxide, and metal–organic frameworks (MOFs) has been shown to significantly enhance gas separation performance beyond the capabilities of neat polymers. MOFs, in particular, have garnered significant interest due to their tunable pore structures, high surface area, and robust thermal and chemical stability—features essential for the selective separation of industrially relevant gas pairs.^3,6,7^

Within the MOF family, zeolitic imidazolate frameworks (ZIFs) stand out for their outstanding gas separation properties. ZIF-67, a cobalt-based MOF, has been extensively studied for its unique combination of properties, including a pore aperture of approximately 3.4 Å, high thermal and chemical stability, and hydrophobic character.^8–10^ These attributes make ZIF-67 particularly suitable for separating gas pairs such as CO_2_/N_2_ and CO_2_/CH_4_.^11,12^ Moreover, the imidazolate linkers in ZIF-67 promote strong interfacial compatibility with polymer matrices, facilitating the development of defect-free MMMs with enhanced gas transport performance. Nonetheless, challenges such as particle agglomeration and structural fragility during membrane fabrication can hinder long-term stability and efficiency.^8,9^

Recent advances in materials science have focused on tailoring the morphology and dispersion of ZIF-67 particles to mitigate these issues. Hydrothermal synthesis, especially with the incorporation of structure-directing agents such as cetyltrimethylammonium bromide (CTAB), has proven effective in controlling the size, shape, and surface chemistry of ZIF-67.^10–13^ Various morphologies—including nanoplatelets, cubes, and hierarchical structures—have been achieved, offering improved compatibility with polymer matrices and enhanced molecular transport properties.^10,11,14–17^ In particular, morphology tailored structures provide increased surface area and improved interfacial interactions, addressing key limitations in MMM fabrication.^12,18^

The choice of polymer matrix also plays a pivotal role in MMM performance. Pebax MH-1657, a block copolymer known for its high CO_2_ permeability and selectivity, has gained traction as an effective host for advanced fillers such as ZIF-67. The combination of Pebax and ZIF-67 has demonstrated promising potential to overcome the traditional permeability–selectivity trade-off observed in conventional polymer membranes.^19,20^ Furthermore, the interaction between ZIF-67 and the Pebax matrix can influence the polymer’s crystallization behavior, thus affecting its mechanical properties and gas transport performance.^21,22^

Among the various ZIFs, ZIF-67 was selected in this study because, compared to the widely studied ZIF-8, it exhibits higher porosity and stronger interactions between CO_2_ molecules and the Co^2+^ sites within its framework, which favor selective CO_2_ transport through membranes^22^. These features, together with its robust thermal and chemical stability, hydrophobic nature, and strong interfacial compatibility with polymer matrices, make ZIF-67 particularly suitable for CO_2_/N_2_ and CO_2_/CH_4_ separations.^8,10,12,22^

Previous studies have reported improved gas separation performance in MMMs incorporating ZIF-67. For instance, Meshkat et al.^22^ showed that Pebax membranes with ZIF-67 exhibited higher CO_2_ permeability and CO_2_/N_2_ selectivity than those with ZIF-8, confirming the strong interaction of Co^2+^ sites with CO_2_ molecules. Feng et al.^21^ demonstrated that morphology-regulated ZIF-67 nanosheets enhanced both permeability and selectivity, while Zhao et al.^27^ observed that hierarchical ZIF-67 structures could intensify CO_2_ separation efficiency. Similarly, Liu et al.^34^ found that MMMs containing ZIF-8-at-ZIF-67 core–shell composites achieved simultaneous improvements in CO_2_ permeability and CO_2_/CH_4_ selectivity. These findings further justify the choice of ZIF-67 as a promising filler for high-performance MMMs.

Building upon these developments, this study investigates the synthesis and integration of morphologically tailored ZIF-67 particles into Pebax MH-1657-based MMMs. The objective is to evaluate how particle size, shape, and surface characteristics influence the structural, thermal, and functional properties of the resulting membranes. By examining synthesis parameters—particularly the role of CTAB as a morphology modulator—this work aims to improve filler dispersion, interfacial compatibility, and membrane stability. The ultimate goal is to develop high-performance MMMs with enhanced gas separation efficiency, contributing to scalable and cost-effective solutions for industrial gas separation applications.

## 2 Experimental Section

### 2.1 Materials

PEBAX MH-1657 copolymer, supplied by Arkema Brazil, has a melting temperature (*T*_m_) of 204 °C and a glass transition temperature (*T*_g_) of −40 °C, as per the manufacturer’s specifications. The reagents used in the synthesis of ZIF-67 were: 2-methylimidazole (MW = 82.10 g/mol, purity 99%), cobalt­(II) acetate tetrahydrate (MW = 249.08 g/mol, purity 98%), and cetyltrimethylammonium bromide (CTAB) (MW = 364.45 g/mol, purity 98%), all purchased from Sigma-Aldrich. Methanol (MW = 32.04 g/mol, purity 98%) was obtained from Êxodo Científica. Distilled and ultrapure water (Milli-Q system) were also used for experimental preparations.

### 2.2 Synthesis of ZIF-67 Particles

ZIF-67 particles were synthesized hydrothermally using a molar ratio of Co^2+^/2-MeIM/H_2_O = 1:32:1800, as described by Yang et al.^43^. The process was carried out in a 150 mL autoclave with 64 mL of water. Two solutions were prepared: (a) 65.36 mmol of 2-methylimidazole dissolved in 32 mL of water, and (b) 2.179 mmol of cobalt­(II) acetate tetrahydrate dissolved in 32 mL of water. In solution (a), cetyltrimethylammonium bromide (CTAB) was added, and the mixture was stirred (∼1800 rpm) until dissolved. Solution (b) was then added under the same stirring conditions, forming a homogeneous emulsion, which was stirred for an additional 5 min. CTAB concentrations ranged from 0.075 wt % to 0.12 wt % (relative to the total mass) to control the morphology, from nanocubes to plate-like structures. The reaction mixture was heated at 140 °C for 24 h, then cooled to room temperature. The product was washed three times with methanol, recovered by centrifugation at 10,000 rpm for 10 min, and dried at 60 °C for 24 h. Finally, the ZIF-67 particles were thermally treated in an inert atmosphere at 300 °C for 150 min to remove residual unreacted compounds.

### 2.3 Membrane Preparation

PEBAX MH-1657/ZIF-67 membranes were prepared using a reflux method under constant stirring in a nitrogen atmosphere. The solution, composed of ethanol and water (70/30), was fractionated, with two-thirds of this solution used to dissolve PEBAX (3 wt %). In a separate step, ZIF-67 nanoparticles at concentrations of 1 and 5 wt % were dispersed in one-third of the total solvent volume using an ultrasonic bath (7Lab, model SSBu-3.8L) for 30 min. After complete dispersion, the ZIF-67 particles were added to the PEBAX solution, and the mixture was stirred for 24 h. The final solution was poured into Teflon Petri dishes and left to rest at ambient temperature for 48 h. The membranes were then dried in a vacuum oven at 50 °C for 24 h to ensure complete removal of solvents. Table 1 presents the compositions and nomenclature of the mixed matrix membranes (PEBAX/ZIF-67) characterized in this study.

**Table 1:** Nomenclature Adopted for MMM/ZIF-67 Samples Synthesized by the Hydrothermal Method

Sample	PEBAX MH-1657 Composition (wt %)	ZIF-67 Composition	CTAB (wt %)	Particle Morphology
RD 1%	3% PEBAX	1% ZIF-67	None	Rhombic Dodecahedron
RD 5%	3% PEBAX	5% ZIF-67	None	Rhombic Dodecahedron
PL 1%	3% PEBAX	1% ZIF-67	0.12%	Plate
PL 5%	3% PEBAX	5% ZIF-67	0.12%	Plate
NC 1%	3% PEBAX	1% ZIF-67	0.075%	Nanocube
NC 5%	3% PEBAX	5% ZIF-67	0.075%	Nanocube

### 2.4 Analysis

The morphology of the mixed matrix membranes (MMMs) was examined using scanning electron microscopy (SEM) with a JEOL JSM 6360-LV microscope, operating at an acceleration voltage of 15 kV, magnification of 75,000x, and a resolution of 500 nm. For sample preparation, the MMMs were cryofractured by immersion in liquid nitrogen for 1 h to obtain cross-sectional images. X-ray diffraction (XRD) analysis was performed to evaluate the crystalline structure of the MMMs and identify any structural changes induced by the incorporation of ZIF-67 nanoparticles. Fourier transform infrared (FTIR) spectroscopy, conducted with a Bruker 70v FTIR equipped with an ATR (Attenuated Total Reflectance) accessory, was used to assess chemical interactions between the polymer matrix and the ZIF-67 fillers.

Differential scanning calorimetry (DSC) was employed to investigate the thermal transitions of the membranes, including the glass transition temperature (*T*_g_), melting behavior, and crystallization. The NETZSCH DSC 200F3 calorimeter was used, with samples initially heated to 120 °C to remove moisture. After cooling to −70 °C, the samples were heated up to 250 °C to eliminate the effects of thermal and processing history. They were then cooled back to −70 °C and heated again to 250 °C at a rate of 10 °C/min, with a nitrogen flow rate of 20 mL/min. The crystallinity (%Xc) of PEBAX MH-1657 was determined from the melting enthalpies using eq 1, and the total degree of crystallinity (%Xt) in the membranes was calculated using eq 2.

$$\%X_{c}={\left[\frac{\Delta H_{m}}{\varphi^{*}\Delta H_{0}}\right]}^{*}100$$ 1

$$\%X_{t}=\frac{\left(\%{X_{cPEO}}^{*}\varphi_{PEO}+\%{X_{cPA}}^{*}\varphi_{PA}\right)}{\varphi_{PEBAX1657}}$$ 2

ΔH _m_ represents the enthalpy associated with the melting peak _(_T _m)_, while ΔH _0_ denotes the enthalpy of fusion for 100% crystalline phases, which are 230 J/g for PA and 166.4 J/g for PEO. The variables ϕ represent the mass fractions of PEO and PA within PEBAX MH-1657 (PEO = 60%; PA = 40%), and ϕ_PEBAX1657 corresponds to the proportion of PEBAX MH-1657 in the mixed matrix membranes, considering the percentage of added particles.^23,24^

### 2.5 Gas Permeation Tests

Gas permeability and ideal selectivity for N_2_, CH_4_, and CO_2_ were measured using a variable volume/constant pressure permeation cell. The experimental setup included a stainless-steel cell connected to a gas supply upstream and a bubble flow meter downstream. Industrial-grade gases were used for testing, which was conducted at 35 °C and at upstream pressures of 10 and 15 bar for each gas. The membrane separation area was 17.34 cm^2^. Downstream flow was monitored until the difference between consecutive measurements fell below 5%. For each membrane sample, two measurements were made with duplicate membranes, except when significant deviations between duplicates required additional testing. Gas permeability was calculated using eq 3, while ideal selectivity was determined using eq 4.

$$P\left[Barrer\right]=\frac{\Delta V}{\Delta t}\left[\frac{cm^{3}}{s}\right]\frac{273\left[K\right]}{T\left[K\right]}\frac{t\left[cm\right]}{A\left[{cm}^{2}\right]\Delta P\left[cmHg\right]}\times 10^{10}$$ 3

$$\alpha_{A/B}=\frac{P_{A}}{P_{B}}$$ 4

Here, $\frac{\Delta V}{\Delta t}$ represents the flow rate measured by the flow meter, T is the testing temperature, t is the membrane thickness, A is the permeation area, ΔP is the pressure difference between upstream and downstream, and P _A_ and P _B_ correspond to the permeabilities of the more and less permeable gases, respectively.

## 3 Results and Discussion

### 3.1 Membrane Characterization

Figure 1 presents the X-ray diffraction (XRD) results for mixed matrix membranes (MMMs) composed of PEBAX MH-1657 and ZIF-67, synthesized using the hydrothermal method. The XRD pattern of pure PEBAX is also included for comparison. The peaks at 2θ = 19° and 2θ = 23.5° correspond to the amorphous poly­(ethylene oxide) (PEO) segments and the semicrystalline polyamide (PA) blocks, respectively.^25,26^ The amorphous halo of PEO is more pronounced in the compositions with 1% RD, 5% PL, and 1% NC. For the 5% RD sample, peaks associated with the (022), (013), and (222) planes of the ZIF structure are observed within this halo, confirming the stability results and linking these particles to improved stability.^21,27^ Despite the low loading (1% to 5%) in all samples, an increase in XRD intensity at approximately 7° is observed, which is attributed to the ZIF structure, particularly the (011) plane.

**Figure 1:**
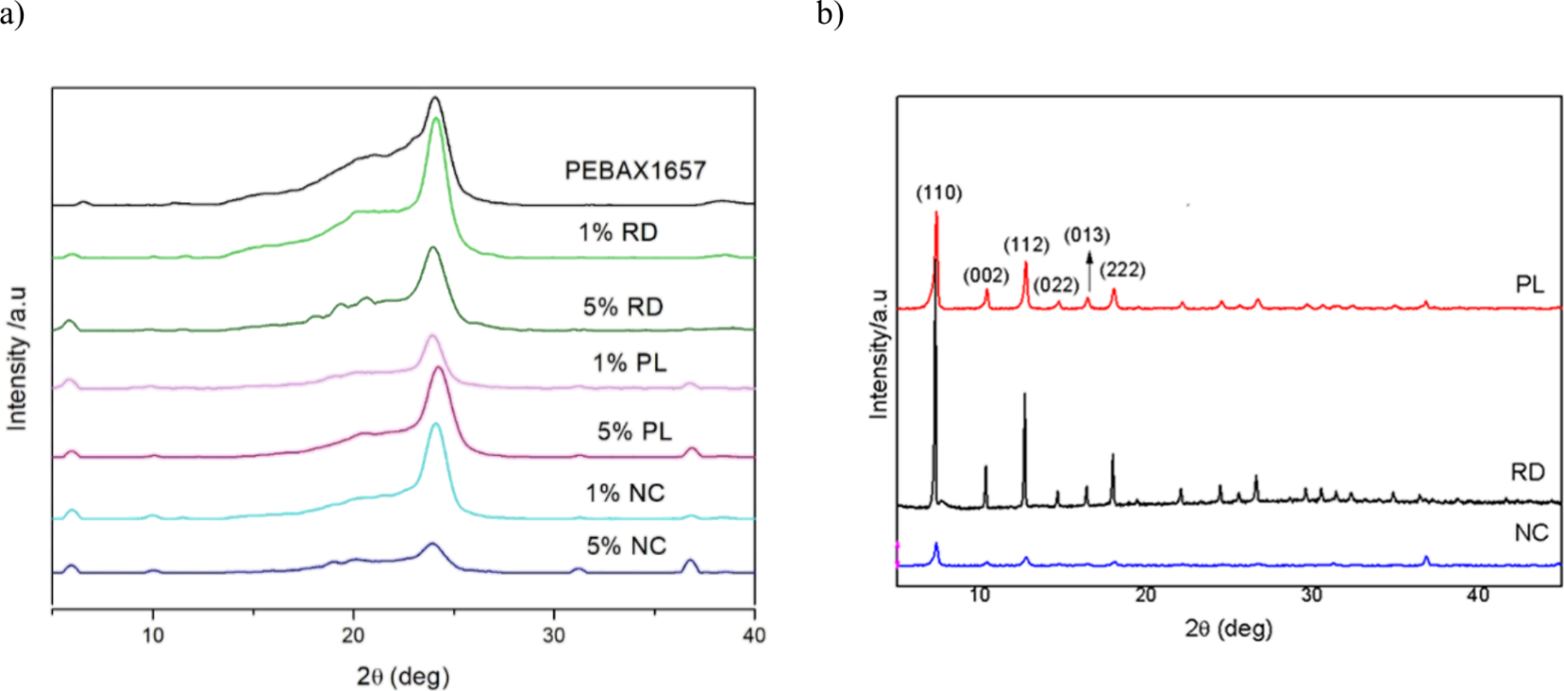
XRD patterns of (a) PEBAX MH-1657/ZIF-67 mixed matrix membranes (MMMs) synthesized by the hydrothermal method and (b) ZIF-67 particles synthesized hydrothermally in the presence of CTAB.

The reduction in the intensity of the polyamide (PA) peak in the MMM samples indicates a significant interaction between the particles and the PEBAX structure, resulting in a decrease in the crystallinity of the PA. This effect is more pronounced in the 5 wt % NC sample. For the MMMs with PL and NC crystals, a peak around 37° was observed, associated with the cobalt structure in the XRD patterns. This peak increased significantly with the loading from 1 wt % to 5 wt % in both morphologies.

In the FTIR-ATR spectra of the membranes (Figure 2), the bands at 1541, 1637, 1730, and 3296 cm^–1^ are associated with NH bending, C = O stretching (HNC = O), C = O stretching (OC = O), and NH stretching, mainly in the polyamide (PA) region. The peak at 1095 cm^–1^ corresponds to COC stretching in the more flexible part of PEBAX, associated with PEO. Additionally, the peak at 1460 cm^–1^ is attributed to sp^3^-CH bending, while the peaks at 2867 and 2935 cm^–1^ are attributed to sp^3^-CH stretching, present in both the rigid and flexible parts of PEBAX MH-1657.^28,29^

**Figure 2:**
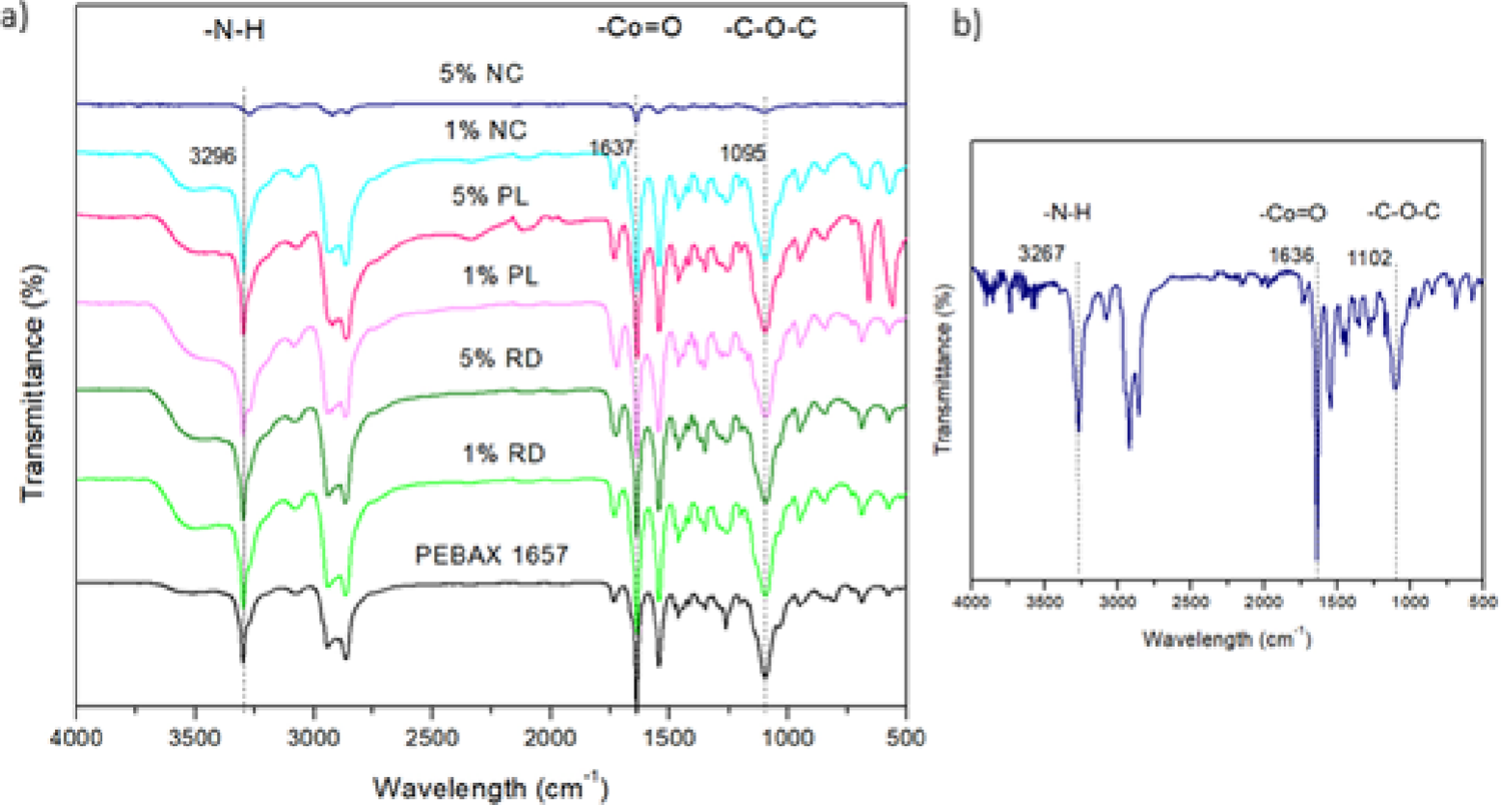
FTIR-ATR spectra of PEBAX MH-1657/ZIF-67 mixed matrix membranes (MMMs) synthesized by the hydrothermal method: (a) pure Pebax and MMMs with 1 and 5 wt % ZIF-67 (RD, PL, and NC morphologies); (b) magnified spectrum of the 5% NC composite.

The morphological and spectroscopic characterizations of ZIF-67 particles with different morphologies have been previously reported by our group in Langmuir^30^. For clarity, the corresponding FTIR spectra and SEM images are shown here in an adapted form with permission from ACS Publications (Figure 3).

**Figure 3:**
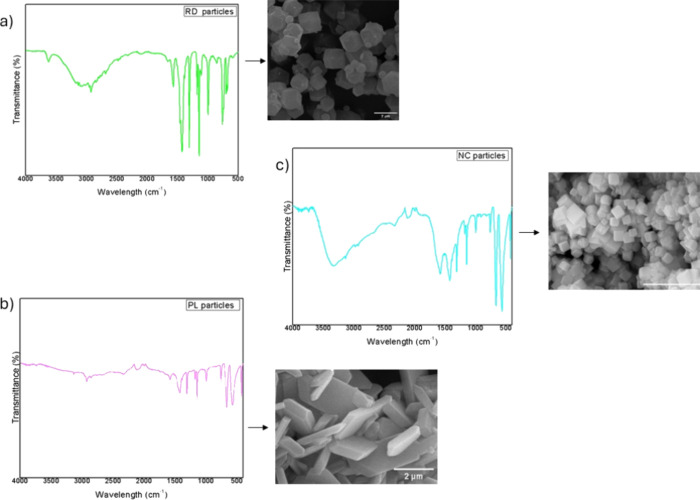
FTIR spectra and SEM images of ZIF-67 particles with different morphologies: plate-like (PL), rod-like (RD), and nanocube (NC). Adapted with permission from ref [^30^]. Copyright 2025 American Chemical Society.

No significant changes were observed in the peaks of the mixed matrix membranes when compared to the pure PEBAX spectrum. Although the 5 wt % NC sample exhibited lower transmittance intensity, an expanded view provided a more accurate depiction of the curves. In the spectra of the MMMs, a significant increase in the bands at 990 cm^–1^ and 764 cm^–1^ was observed, indicating the incorporation of particles into the membrane. For the 5 wt % particle composite, a noticeable shift in the baseline to the peak at 764 cm^–1^ was observed, which is associated with the out-of-plane bending of the MeIM ring. In summary, except for the 5 wt % NC composite, the addition of particles did not cause significant changes when compared to pure PEBAX, and the increase in intensity of peaks related to the ZIF structure indicated good adhesion to the membrane.

The differential scanning calorimetry (DSC) analysis (Table 2) shows that ZIF-67 incorporation modulates the thermal behavior of PEBAX MH-1657 in a morphology- and loading-dependent manner. The *T*_g_ decreased from – 50.5 °C (neat) to as low as – 65.4 °C (5 wt % RD), reflecting enhanced chain mobility linked to reduced ordering in the PA-rich phase. The PEO melting temperature remained essentially stable (≈14–18 °C), except in the 1 wt % PL sample where it was undetectable. These results indicate that ZIF-67 primarily influences segmental dynamics and PA ordering, while PEO crystallinity is only selectively affected.^23,31,32^ .

**Table 2:** Thermal Properties of PEBAX MH-1657/ZIF-67 MMMs Synthesized By the Hydrothermal Method

Sample	*T*_g_ (°C)	T_m,_ PEO (°C)	*T*_m_, PA (°C)	*X*_c,_ PEO (%)	Xc, PA (%)	Xt (%)
PEBAX MH-1657	–50.5	13.5	204.1	18.6	30.9	23.5
1% RD	–52.7	18.3	205.3	25.9	22.6	24.8
5% RD	–65.4	17.4	204.3	22.1	25.3	24.9
1% PL	–53.6	-	181.6	-	28.5	11.5
5% PL	–52.7	18.00	203.2	22.6	27.3	25.5
1% NC	–57.5	13.9	211.3	21.8	37.5	28.4
5% NC	–60.1	15.9	204.2	22.9	17.5	21.8

In contrast, the PA segment exhibited more substantial variations in the melting temperature (*T*_m_). For instance, the 1 wt % PL sample showed a decrease in *T*_m_ from 204.1 to 181.6 °C, whereas the 1 wt % NC sample demonstrated an increase in *T*_m_ to 211.3 °C. These findings emphasize the role of ZIF-67 dispersion and concentration on the crystalline structure of the PA segment, indicating that nanoparticle–polymer interactions can either decrease or enhance PA crystallinity depending on morphology and distribution^33^.

The incorporation of ZIF-67 also modified the crystallinity of the polymer matrix in a morphology-specific fashion. For PEO, the 1 wt % RD sample showed a significant increase in crystallinity (25.9%), suggesting that ZIF-67 nanoparticles promoted a more ordered PEO structure. Conversely, the PA segment generally exhibited reduced crystallinity, except for the 1 wt % NC sample, which achieved the highest PA crystallinity (37.5%). These results highlight that well-dispersed ZIF-67 particles facilitate the formation of ordered crystalline domains, while aggregation tends to disrupt polymer organization^22^.

X-ray diffraction (XRD) analysis further supports these findings, showing sharper and more intense ZIF-67 peaks in this study, indicative of higher crystallinity and better nanoparticle alignment within the matrix. In the prior solvothermal study, weak and diffuse XRD peaks were reported, reflecting poor particle dispersion and crystallinity^33^.

Additionally, the improved synthesis conditions influenced the membranes’ thermal properties. For instance, this study observed a more pronounced reduction in *T*_g_, indicative of increased polymer flexibility due to the hierarchical ZIF-67 structure. This effect is particularly advantageous for applications requiring enhanced membrane flexibility and thermal efficiency, such as CO_2_ separation technologies.

The formation of a hierarchical ZIF-67 structure via hydrothermal synthesis was pivotal for achieving the observed improvements. Enhanced dispersion, particle–matrix adhesion, and crystalline organization collectively contributed to superior thermal and structural properties, critical for optimizing membrane performance in practical applications like CO_2_ capture, where both thermal stability and mechanical integrity are essential.

### 3.2 Gas Permeation Properties of PEBAX MH-1657/ZIF-67 Membranes

The incorporation of hierarchical ZIF-67, with varying morphologies and concentrations, into mixed matrix membranes (MMMs) significantly impacted the CO_2_ permeation properties and selectivity for CO_2_/CH_4_ and CO_2_/N_2_ pairs. The results, shown in Figure 4, revealed substantial variations in CO_2_ permeability and CO_2_/CH_4_ selectivity, depending on the morphology of ZIF-67 and its concentration in the membranes.

**Figure 4:**
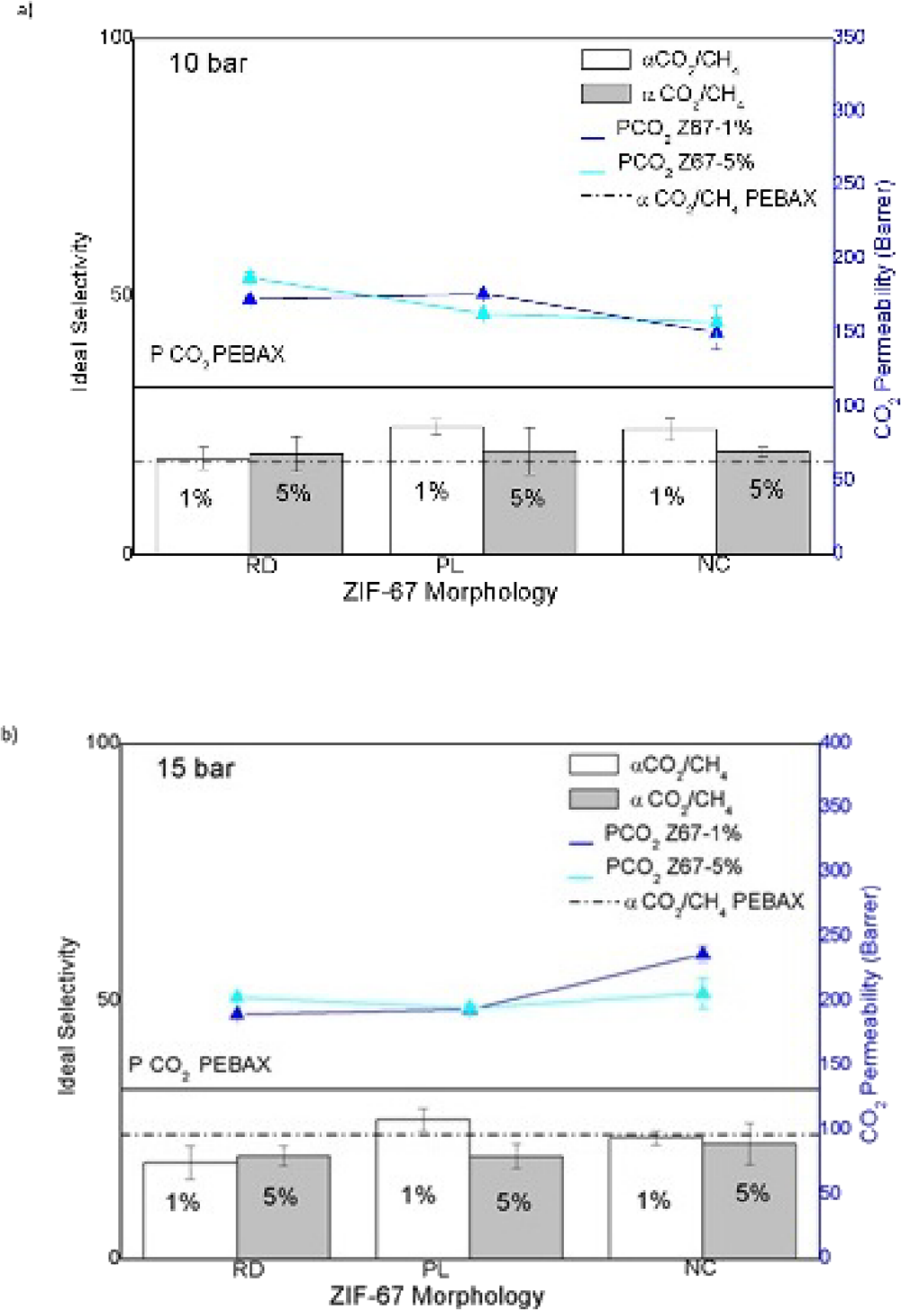
ZIF-67 concentration as a function of CO_2_ permeability and ideal CO_2_/CH_4_ selectivity at a) 10 bar and b) 15 bar.

For membranes with RD, the introduction of 1 wt % ZIF-67 resulted in a 7.9% increase in CO_2_ permeability, without significantly altering selectivity. This can be attributed to the structure of ZIF-67, which limits gas diffusion, acting as an internal barrier that restricts the passage of gases like CH_4_ while allowing more efficient CO_2_ diffusion. In membranes with PL morphology, the same ZIF-67 concentration led to a 7.5% decrease in CO_2_ permeability, but it maintained the selectivity for the CO_2_/CH_4_ pair, with a selectivity value around 25. This behavior is explained by the molecular sieving effect, where ZIF-67 particles form internal barriers for larger gases like CH_4_, facilitating the easier passage of CO_2_.^18,34^ Hierarchical ZIF-67 structures, with optimized microstructures, show great effectiveness in selective gas separation, reinforcing the potential of ZIF-67 as a key material for CO_2_ separation^18,27,34^

For NC morphology membranes at 5 wt % ZIF-67, both CO_2_ and CH_4_ permeabilities increased; however, this was accompanied by a lower selectivity compared to the PL configuration at 1 wt %. Nanocube structures create less restrictive pathways that facilitate CH_4_ diffusion, resulting in reduced selectivity. This behavior is expected since nanocube structures offer easier passage for smaller gases like CH_4_ without an effective molecular barrier to restrict it.^21,27^ Tests conducted at 15 bar confirmed these trends; the PL configuration with 1 wt % ZIF-67 maintained ideal selectivity while in RD membranes, increasing the ZIF-67 concentration from 1 wt % to 5 wt % resulted in a 6.5% increase in CO_2_ permeability without compromising selectivity. This suggests that the uniform distribution of particles in the RD configuration facilitates CO_2_ permeation without undermining selectivity.^7,22^

In comparison to pure PEBAX membranes, MMMs with tuned ZIF-67 morphologies exhibited significant improvements in both permeation properties and selectivity. Pure PEBAX MH-1657 displayed a CO_2_ permeability of 114 Barrer and a selectivity of 18 at 10 bar. The best results were morphology-dependent: PL 5% achieved the highest CO_2_/N_2_ selectivity (110 at 10 bar), PL 1% the highest CO_2_/CH_4_ selectivity (27 at 15 bar), and NC 1% the highest CO_2_ permeability (236 Barrer at 15 bar). At 15 bar, however, most MMMs with ZIF-67 experienced a decrease in selectivity, except for the 5 wt % PL configuration, which showed an 11% improvement over pure PEBAX. This behavior indicates that increased pressure can enhance the permeability of gases like CH_4_, particularly in less restrictive morphologies, which lowers overall selectivity.^22,35^

Overall, increasing the concentration of ZIF-67 resulted in a substantial improvement in CO_2_ permeability, particularly at 15 bar where higher pressures favor CO_2_ flow and optimize separation efficiency. Additionally, the CO_2_/CH_4_ selectivity was also enhanced by the higher concentration of ZIF-67; indicating that this hierarchical material makes membranes more effective at separating CO_2_ from CH_4_ under high pressures, essential for industrial gas separation processes such as CO_2_ capture systems^36^.

In Figure 5, the analysis of permeation properties for the CO_2_/N_2_ pair followed a similar trend to that observed for the CO_2_/CH_4_ pair. At 15 bar, CO_2_ permeability was significantly higher; suggesting that increased pressure favored CO_2_ separation. Similarly, CO_2_/N_2_ selectivity also improved with the presence of ZIF-67, demonstrating its effectiveness in both CO_2_/CH_4_ and CO_2_/N_2_ separations.^22,35,37^

**Figure 5:**
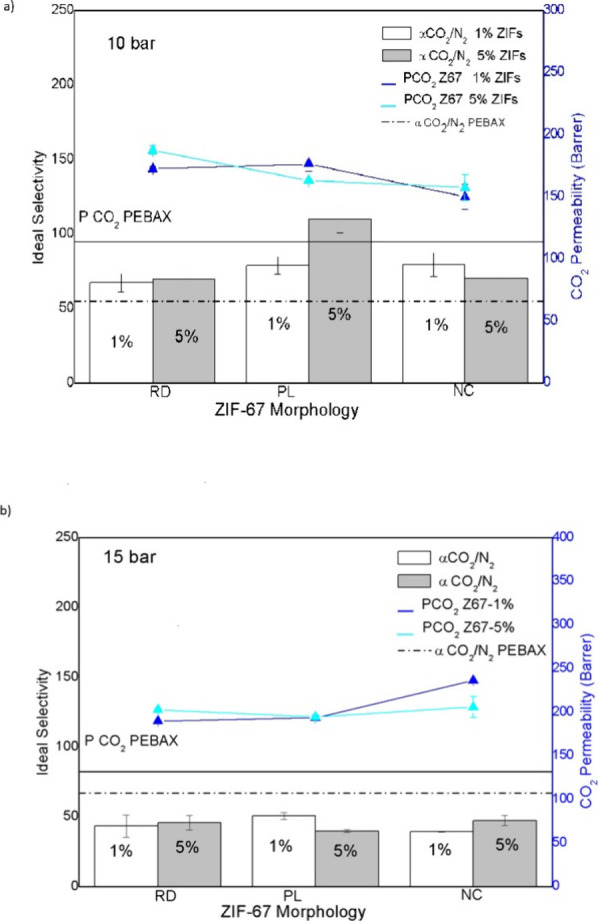
ZIF-67 concentration as a function of CO_2_ permeability and ideal CO_2_/N_2_ selectivity at a) 10 bar and b) 15 bar.

When comparing RD membranes with 5 wt % ZIF-67 at 10 bar to those with a PL configuration at 5 wt %, the highest CO_2_ permeability was observed along with a significant increase in N_2_ permeability, 45% higher than pure PEBAX membranes. The RD configuration at 1 wt % showed a remarkable increase of 69% in N_2_ permeability; and at 15 bar, the NC configuration at 1% achieved the largest increase in N_2_ permeability (69%). The combination of configurations, 5 wt % PL and 1 wt % RD, showed increases in N_2_ permeability of 66.5% and 48.14%, respectively. These results indicate that ZIF-67 can be manipulated to optimize not only the separation of CO_2_ but also other gases such as N_2_; which is relevant in gas purification processes and air separation.^38–40^

Among ZIF materials, ZIF-67 stands out due to its higher porosity compared to ZIF-8, attributable to the substitution of Zn with Co. This modification enhances interactions between CO_2_ and Co sites within the ZIF-67 structure, facilitating selective CO_2_ transport, while weaker interactions with CH_4_ and N_2_ arise from their lower polarity. These stronger interactions make ZIF-67 a promising candidate for CO_2_ separations.^36,41^

Furthermore, tuned morphologies of ZIF-67 can strengthen these polar interactions with CO_2_, enabling its preferential passage while restricting diffusion of larger or less polar gases such as CH_4_ and N_2_. This behavior has been consistently observed in separations involving both CO_2_/CH_4_ and CO_2_/N_2_ mixtures, underscoring the ability of ZIF-67 to optimize gas separation through a combination of molecular interactions and morphological control.^39–42^

Table 3 summarizes the gas separation performance of PEBAX/ZIF-67 membranes prepared in this work (Table 3a) and representative studies from the literature (Table 3b). As shown in Table 3a, the effect of morphology (NC, PL, RD) and filler loading (1 and 5 wt %) under different pressures (10 and 15 bar) led to CO_2_ permeabilities up to 236 Barrer, with CO_2_/N_2_ and CO_2_/CH_4_ selectivities reaching 110 and 27, respectively. Compared with these results, the literature data in Table 3b confirm that most studies have focused on ZIF-8 or unmodified ZIF-67 fillers, with Pebax 1657 being the most commonly used polymer matrix. Zhao et al.^27^, Liu et al.^43^, Nobakht & Abedini^31^, Zhu et al.^36^, and Salehi & Raisi^44^ reported improved CO_2_ separation performance with hierarchical ZIF-67, core–shell ZIF composites, or alternative fillers, but none employed CTAB-modified ZIF-67. In the case of Yang et al.^19^, which investigated CTAB-assisted morphological control of ZIF-8, only the C_3_H_6_/C_3_H_8_ gas pair was tested. Therefore, the results presented here provide the first direct evidence of the role of CTAB-assisted morphological modification of ZIF-67 in PEBAX, representing an original contribution to the advancement of MMMs for CO_2_ separation.

**Table 3:** (**a**) Gas Separation Performance of PEBAX/ZIF-67 Membranes (This Work). (**b**) Gas Separation Performance of Pebax-Based MMMs Reported in the Literature^a^

Sample	Pressure (bar)	P_CO_2__ (Barrer)	α _CO_2_/N_2__	α _CO_2_/CH_4__
RD 1%		172.6	67.3	18.7
RD 5%		187.5	70.0	19.5
PL 1%		176.4	78.9	24.8
PL 5%	10	163.2	110	20.2
NC 1%		150	79.5	24.4
NC 5%		157	70.0	20.0
RD 1%		190	43.5	18.7
RD 5%		203	46	20
PL 1%	15	193.6	50.0	27.0
PL 5%		194.4	40.0	20.0
NC 1%		236.0	39.5	23.5
NC 5%		206.0	47.4	22.3

Reference	MOF/Modifier	Polymer matrix	Pressure (bar)	P_CO_2__ (Barrer)	α _CO_2_/N_2__	α _CO_2_/CH_4__
[^27^]	Leaf-like hierarchical ZIF-67	Pebax MH-1657	10	160	75	—
[^31^]	Pebax/maltitol/ZIF-8 (5 wt %)	Pebax MH-1657	10	170	69	27
[^36^]	hZIF-L	Pebax MH-1657	10	110	—	18–20
[^43^]	Core–shell ZIF-8@GO	Pebax MH-1657	1	173	62	12
[^44^]	ZIF-67 (16 wt %)	Pebax MH-2533	4	190.5	39.7	22.5

a Note: Data from refs 27, 31, 36, 43, 44.

Figure 6 compares the performance of mixed matrix membranes (MMMs) synthesized via hydrothermal methods with pure PEBAX MH-1657 membranes for the CO_2_/CH_4_ pair. For the CO_2_/N_2_ system, the trade-off plot includes MMMs developed in this study alongside reference MMMs produced by the same group using solvothermal synthesis. Reference data for pure PEBAX and solvothermally synthesized MMMs were taken from prior work^33^.

**Figure 6:**
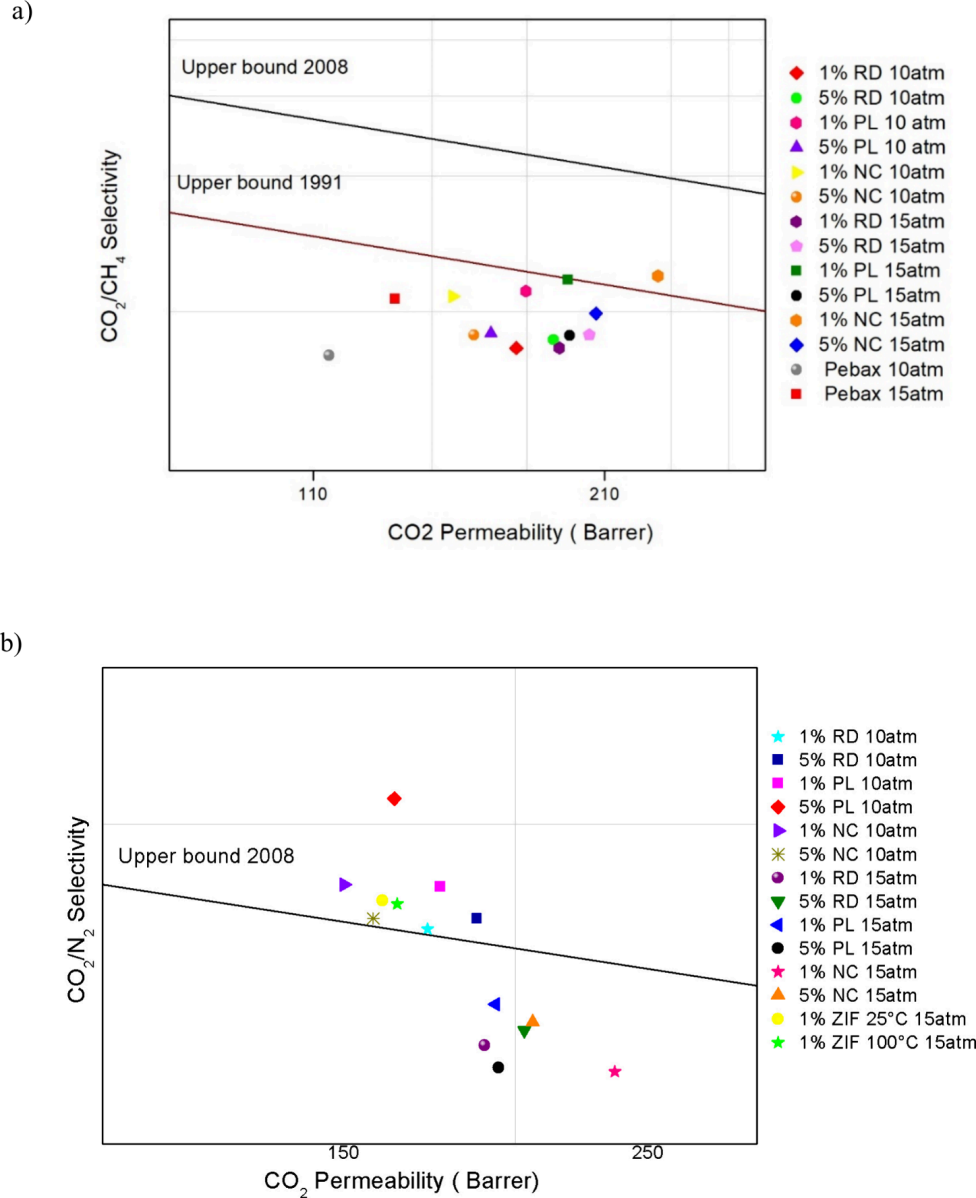
Performance of PEBAX MH-1657/ZIF-67 MMMs (hydrothermal method) on the trade-off plot at 10 and 15 bar, compared with (a) neat PEBAX MH-1657 membranes for the CO_2_/CH_4_ gas pair; (b) MMMs prepared via hydrothermal and solvothermal methods for the CO_2_/N_2_ gas pair.

For CO_2_/CH_4_ separation, the hydrothermally synthesized MMMs demonstrate enhanced permeability and selectivity relative to neat PEBAX membranes, positioning them toward the upper right quadrant of the trade-off plot. However, none exceed the Robeson 2008 upper bound^45^ In contrast, for CO_2_/N_2_ separation, MMMs tested at 15 bar fall below the upper bound, but at 10 bar, all MMMs with 1% or 5 wt % ZIF-67, regardless of crystal morphology (RD, PL, or NC), surpass this limit. Except for the 1 wt % NC sample, these MMMs also outperform solvothermally synthesized counterparts from earlier studies. This performance trend is attributed to the low loading of ZIF-67, which limits the formation of continuous gas transport pathways, as well as to the inherent tendency of ZIFs to adsorb and transport gas molecules larger than their nominal pore apertures. The pressure effect showed a distinct behavior, which can be explained by the presence of particles with different morphologies and larger sizes, leading to a reduction in selectivity due to increased permeability of CH_4_ and N_2_.^40,46^

Finally, crystallinity and glass transition temperature play crucial roles in membrane permeation properties. Hierarchical ZIF-67 altered crystallinity within polymer phases, influencing molecular chain mobility and consequently affecting CO_2_ permeability.^18,27,34^ In both RD and PL morphologies, ZIF-67 significantly impacted crystallinity, improving both permeability and selectivity. For instance, RD configurations containing 5 wt % ZIF-67 showed increased CO_2_ permeability linked to reduced crystallinity within polyamide, favoring chain mobility.^7,22^ In contrast, PL configurations exhibited increased crystallinity within PEO, restricting CO_2_ diffusion but enhancing selectivities for both CO_2_/N_2_ and CO_2_/CH_4_.^22,47^

Precise control of crystallinity and *T*_g_, combined with the hierarchical structure of ZIF-67, proved essential for optimizing the balance between permeability and selectivity.^18,27^ The comparison between solvothermal and hydrothermal synthesis methods revealed significant differences in the characteristics of ZIF-67 particles, which directly impacted the properties of the resulting membranes. ZIF-67 particles synthesized via the solvothermal method, as described in a previous study by our group, exhibited greater stability, smaller crystal size, and larger surface area compared to those produced through hydrothermal synthesis^32^. In contrast, the present study demonstrates the superior performance of hydrothermally synthesized PEBAX/ZIF-67 MMMs, particularly regarding particle dispersion and initial improvements in permeability (+26%) and selectivity (+39%) relative to neat PEBAX membranes. Nevertheless, solvothermally synthesized ZIF-67 structures showed greater structural stability under elevated operating pressure (15 bar), maintaining consistent selectivity values in conditions where hydrothermal samples exhibited partial performance loss^33^.

An important aspect worth noting is that solvothermal methods do not generate hierarchical structures like hydrothermal methods, resulting instead in simpler yet more uniform particles which enhance both PEBAX membrane permeability and selectivity.^48,49^ Conversely, hydrothermally synthesized particles typically show smaller surface areas along with greater instability within aqueous environments, particularly noticeable within plate-like or nanocube configurations.

The structural instability associated with hydrothermally synthesized ZIF-67 particles emerges as a critical consideration when evaluating membrane performance under variable pressure conditions. Studies indicate that hydrothermal structures ideal for selective CO_2_ separation may undergo expansion or loss of organization under high pressures, as evidenced during membrane tests conducted at elevated pressures (15 bar). Such deformation can lead toward increased permeabilities concerning larger molecules like CH_4_, thereby compromising overall membrane selectivity.^31,34^

In contrast, solvothermal synthesized ZIF-67 maintains structural integrity even under high pressures, preserving its capability to selectively filter out larger unwanted molecules while allowing efficient transport of targeted gases like carbon dioxide.^49–51^ Although solvothermal methods yield nonhierarchical structures, they result in stable frameworks that ensure effective porosity remains intact even amidst elevated pressures. This promotes optimal conditions conducive to enhanced carbon dioxide permeation without substantial losses in overall selective performance.^34,50,52^

## 4 Conclusions

The characterization of PEBAX MH-1657/ZIF-67 mixed matrix membranes (MMMs) synthesized via the hydrothermal method revealed significant changes in their structural and thermal properties, which influenced gas permeation performance at pressures of 10 and 15 bar. X-ray diffraction analysis confirmed strong interactions between ZIF-67 and the PEBAX matrix, characterized by a reduction in polyamide crystallinity and increased flexibility of the polymer matrix. Differential scanning calorimetry further supported these findings, showing a decrease in the glass transition temperature as ZIF-67 concentration increased, which enhanced CO_2_ permeability and affected the permeability-selectivity balance. Fourier-transform infrared spectroscopy spectra confirmed nanoparticle incorporation and underscored the importance of nanoparticle–polymer interactions in optimizing membrane properties.

Membranes with different ZIF-67 morphologies demonstrated improvements in CO_2_ permeability, with variations in selectivity. The NC 1% membrane at 15 bar achieved the highest CO_2_ permeability (236 Barrer), while the PL 5% membrane at 10 bar exhibited the highest CO_2_/N_2_ selectivity (110), and the PL 1% membrane at 15 bar reached the highest CO_2_/CH_4_ selectivity (27). The RD morphology enhanced CO_2_ permeability without compromising selectivity. These results highlight that the best performance depends on morphology and operating conditions.

At 15 bar, CO_2_ permeation was favored; however, the increased permeability of other gases resulted in reduced selectivity. This behavior was particularly evident in RD membranes with higher ZIF-67 concentrations, emphasizing the importance of pressure and composition control in optimizing gas separation performance. ZIF-67 also improved CO_2_/CH_4_ and CO_2_/N_2_ selectivity, which are essential for effective CO_2_ capture. Variations in crystallinity and *T*_g_ across morphologies emphasized the role of molecular design in tailoring separation performance.

In conclusion, PEBAX MH-1657/ZIF-67 membranes incorporating hierarchical ZIF-67 structures show significant potential for CO_2_ separation. The morphology of ZIF-67, its concentration within the membranes, and the control of synthesis and pressure conditions are critical for optimizing performance in industrial applications. The hydrothermal synthesis method played a key role in improving particle dispersion and enhancing permeability–selectivity performance. Nevertheless, solvothermal synthesis yielded more pressure-resistant structures, ensuring higher stability at 15 bar. Therefore, the choice between hydrothermal and solvothermal approaches should be guided by the intended application: hydrothermal synthesis maximizes initial gas separation performance under moderate pressures, while solvothermal synthesis ensures structural robustness under elevated pressures.
